# Pathological CMR findings and their clinical value in patients with high grade ventricular arrythmias without previously known cardiac conditions

**DOI:** 10.1186/1532-429X-13-S1-O42

**Published:** 2011-02-02

**Authors:** Daniel Thomas, Claas P Naehle, Darius Dabir, Hans Schild, Georg Nickenig, Victor A Ferrari, Jutta Weisser-Thomas

**Affiliations:** 1 University of Bonn, Bonn, Germany; 2 University of Pennsylvania, Philadelphia, PA, USA

## Introduction

Recent studies have demonstrated the ability of CMR to identify the anatomic correlate of high grade ventricular arrhythmia (VA) in HCM and CHD patients. However, little is known about the value of CMR in patients with VAs, without previously known cardiac conditions.

## Purpose

To investigate the value of CMR for detection of underlying heart disease and arrhythmogenic substrate in patients with VAs.

## Methods

Patients with VAs, without known cardiac condition were included (n=71). CMR, echocardiography and 12-lead ECGs were performed in all patients. Most patients underwent electrophysiologic testing (EPS). CMR findings were correlated with ECG and/or EPS findings. A morphological substrate was defined as 1) underlying cardiac condition detected by CMR, known to cause associated arrhythmia, or 2) presence of an electro-anatomic correlate, if the location of a structural abnormality/scar tissue, matched the origin of the VA as defined by ECG and/or EPS. A positive CMR finding was defined as clinically relevant if 1) it resulted in a new diagnosis, or 2) a change of treatment, or 3) additional diagnostic procedures.

## Results

Pathological CMR findings were found in 42 patients. The main findings were wall motion abnormalities (n=22), ventricular hypertrophy (n=10), dilatation (LV or RV or atrium, total=28), wall thinning (n=3), pericardial and/or pleural effusion (n=6), myocardial scar/structural abnormalies (n=14).

Based on CMR a diagnosis of heart disease was established in 19 patients (27%; DCM n = 3, HCM n = 1, criteria for ARCM n = 2, post-myocarditis scar n = 7, post-myocardial infarction scar n = 2, myocarditis n = 4). In 15/71 patients (21%) the definite diagnosis was obtained by CMR exclusively. All CMR based diagnoses were classified as morphological substrates. In 8 patients the location of the relevant CMR finding (myocardial scar) directly matched the origin of the VA as defined by ECG or EPS. In none of these patients the relevant finding was detected by echocardiography. The diagnoses resulted in a change of treatment in 11 patients (15%) and a change of treatment plus additional diagnostic procedures in 8 patients (11%).

## Conclusions

This is the first study revealing the value of CMR for detection of the morphological substrate and/or underlying cardiac condition in patients with VAs, without any previously known cardiac disease. The high incidence of clinically relevant CMR findings raises the question, whether CMR should routinely be performed in those patients.

